# Development and Reliability Testing of a Health Action Process Approach Inventory for Physical Activity Participation among Individuals with Schizophrenia

**DOI:** 10.3389/fpsyt.2014.00068

**Published:** 2014-06-10

**Authors:** Kelly P. Arbour-Nicitopoulos, Markus Duncan, Gary Remington, John Cairney, Guy E. Faulkner

**Affiliations:** ^1^Faculty of Kinesiology and Physical Education, University of Toronto, Toronto, ON, Canada; ^2^Bloorview Research Institute, Toronto, ON, Canada; ^3^Schizophrenia Program, Centre for Addiction and Mental Health, Toronto, ON, Canada; ^4^Faculty of Medicine, University of Toronto, Toronto, ON, Canada; ^5^Department of Medicine, McMaster University, Hamilton, ON, Canada; ^6^Department of Psychiatry and Behavioural Neuroscience, McMaster University, Hamilton, ON, Canada

**Keywords:** schizophrenia, physical activity, determinants, theory-based, reliability testing, measurement

## Abstract

Individuals with schizophrenia tend to have high levels of cardiovascular disease and lower physical activity (PA) levels than the general population. Research is urgently required in developing evidence-based behavioral interventions for increasing PA in this population. One model that has been increasingly used to understand the mechanisms underlying PA is the health action process approach (HAPA). The purpose of this study was to adapt and pilot-test a HAPA-based inventory that reliably captures salient, modifiable PA determinants for individuals with schizophrenia. Initially, 12 outpatients with schizophrenia reviewed the inventory and provided verbal feedback regarding comprehension, item relevance, and potential new content. A content analysis framework was used to inform modifications to the inventory. The resultant inventory underwent a quantitative assessment of internal consistency and test–retest reliability. Twenty-five outpatients (*M*_age_ = 41.5 ± 13.5 years; 64% male) completed the inventory on two separate occasions, 1 week apart. All but two scales showed good internal consistency (Cronbach’s α = 0.62–0.98) and test–retest correlations (*r*s = 0.21–0.96). Preliminary assessment of criterion validity of the HAPA inventory showed significant, large-sized correlations between behavioral intentions and both affective outcome expectancies and task self-efficacy, and small to moderate correlations between self-reported minutes of moderate-to-vigorous PA and the volitional constructs of the HAPA model. These findings provide preliminary support for the reliability and validity of the first-ever inventory for examining theory-based predictors of moderate-to-vigorous PA intentions and behavior among individuals with schizophrenia. Further validation research with this inventory using an objective measure of PA behavior will provide additional support for its psychometric properties within the schizophrenia population.

## Introduction

Good physical health is a realistic goal for people with schizophrenia, and lifestyle programs that consider physical activity (PA) are essential (1). The potential for recovery from schizophrenia, and reintegration into the community, is considered multi-factorial and extends beyond symptomatic remission – quality of life for those with schizophrenia also includes physical health (2). Life expectancy is reduced by 20 years in schizophrenia and this is primarily due to cardiovascular disease (CVD) (3, 4). PA reduces CVD risk, however participation levels are significantly lower among people with schizophrenia compared to the general population (5). Research is urgently required in developing evidence-based behavioral interventions for increasing PA that are tailored to this population (6).

Within the general population, researchers have identified modifiable, theory-based predictors of PA that have formed the basis for interventions aimed at changing PA behavior (7). Several cross-sectional studies have identified self-efficacy (8–10), social support (9–11), perceived benefits (9, 12), and intentions (8) to be consistent, modifiable theory-based PA correlates among persons with severe mental illness (SMI) such as schizophrenia, major depression, and bipolar disorder. While a correlational relationship suggests an association exists between two factors, it does not imply causality. Rather, a causal relationship indicates that changes in one variable are systematically followed with changes in another variable, which is necessary for identifying the most important PA determinants to target in future interventions (13). Given the lack of prospective theory-based PA research in the schizophrenia population, the relative importance of potential PA determinants specific to persons with schizophrenia still needs to be identified (14) – particularly given the central role motivational deficits play in this disorder (15), which may be different to other populations.

**Figure 1:**
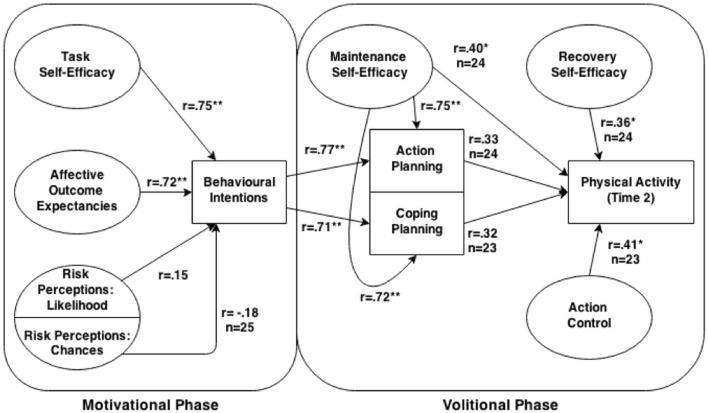
**Diagram of the health action process approach model with obtained Pearson correlations between relevant paths**. Unless otherwise specified, for all correlations within the volitional phase *n* = 26, and *n* = 25 within the motivational phase.

One model that has been increasingly used to understand the mechanisms underlying PA behavior is the health action process approach (HAPA) (16, 17). The HAPA (Figure 1) distinguishes between two phases of behavior change (18), where different social–cognitive predictors may emerge. The pre-intentional motivation phase captures a set of beliefs that are predictive of one’s intention to perform a specific behavior. These pre-intentional beliefs have been identified as *risk perceptions* [perceived susceptibility to a health threat; (19)], *outcome expectancies* [subjective beliefs that particular courses of action will ultimately produce certain desired outcomes; (20)], and *task self-efficacy* [confidence in one’s ability to perform a specific action; (21)]. People in the motivational phase are labeled as pre-intenders. The second, post-intentional volition phase focuses on the self-regulatory strategies needed to plan, initiate, and maintain the behavior. These post-intentional self-regulatory strategies are *action planning* [forming concrete plans which specify when, where, and how an intention or goal will be translated into action; (22)], *coping planning* [planning that involves pairing of anticipated barriers with self-regulatory strategies; (23)], *action control* [processes used to manage action sequences and maintaining long-term behavior change; (24)], *maintenance self-efficacy* [confidence in one’s ability to perform the behavioral task under challenging situations; (25)], and *recovery self-efficacy* [confidence in one’s ability to resume the behavioral task after a setback; (17)]. Individuals in the early (pre-actional) volition phase have the intention to act, but still remain inactive (intenders), while those in the later (actional) volition phase have initiated the intended action (actors) (26). Other barriers and resources are posited to affect intentions, planning, and behavioral engagement, thus having a dynamic influence throughout the behavior change process.

Contrary to traditional theories and models of motivated behavior which focus almost exclusively on behavioral adoption [e.g., Ref. (27, 28)], the HAPA framework includes both pre- and post-intentional factors of the behavior change process. In the pre-intentional (motivation) phase, risk perceptions, outcome expectancies, and task self-efficacy are proposed to be important factors to target for increasing behavioral intentions in persons who are unmotivated. Meanwhile, the HAPA also provides researchers with specific factors to target in the post-intentional (volition) phase (i.e., action and coping planning, action control, maintenance, and recovery self-efficacy), where behavioral initiation and maintenance are of importance. These post-intentional, self-regulatory skills are essential for the promotion of independent PA behavior in populations that must leave the structure of formal rehabilitation (29–31). These HAPA constructs therefore, would be appropriate to target in persons with schizophrenia to assist them with their transition from clinical to community-based PA, thus reducing reliance on health services to deliver support indefinitely.

A second benefit of using the HAPA model over other theories and models of motivated behavior is the focus on phase-specific self-efficacy beliefs. Self-efficacy is a robust predictor of behavior change in a variety of situations (20). However, the strength of association has been shown to vary as a function of the type of self-efficacy (25). According to Schwarzer (16), different tasks must be mastered during the course of behavior change, each of which requiring different types of self-efficacy beliefs. As such, the HAPA includes three types of self-efficacy beliefs (i.e., task, maintenance, and recovery). This distinction between phase-specific self-efficacy beliefs makes the HAPA a useful framework for predicting both intentions and behavior in a variety of domains [e.g., Ref. (28, 32–35)] and settings [e.g., Ref. (23, 31, 36–38)] of behavior change, using prospective designs of both short (35) and longer (39) durations. Furthermore, the HAPA framework is useful for developing tailored lifestyle interventions for people with chronic illness and disability where a lack of motivation and low self-efficacy are common barriers to behavior change [e.g., Ref. (23, 31, 36, 38, 40)]. In sum, the HAPA’s utility for developing tailored health behavior interventions in a variety of populations suggests that it would be an appropriate theoretical framework for promoting PA in the schizophrenia population. However, no research has examined the utility of the HAPA framework for predicting PA within the schizophrenia population.

Given the atheoretical nature of PA interventions so far reported in the literature among individuals with schizophrenia (41), we are conducting a two-phase program of research examining the determinants of PA using the HAPA framework. The primary purpose of the first phase, and the current study being reported here, is to examine the internal consistency and test–retest reliability of a HAPA-based PA inventory among a sample of individuals with schizophrenia. A secondary purpose is to examine the relationships between the HAPA motivational phase predictors (i.e., risk perceptions, outcome expectancies, and task self-efficacy) and behavioral intentions and between the HAPA volitional phase predictors (i.e., intentions, maintenance self-efficacy, action planning, coping planning, action control, and recovery self-efficacy) and PA behavior. In the second future phase, we will test how well the HAPA framework predicts objectively measured PA behavior among a larger sample of individuals with schizophrenia. Combined, this knowledge will provide a framework on which to build PA interventions, and to evaluate the social–cognitive constructs potentially mediating PA behavior change.

## Methods and Materials

### Participants

All participants were required to have a diagnosis of schizophrenia or schizoaffective disorder as described by the *Diagnostic and Statistical Manual of Mental Disorders* [4th ed., text rev.; DSM-IV-TR; (42)]. In line with the Canadian Physical Activity Guidelines for Adults (43), participants had to be between the ages of 18 and 64, and had to be outpatients or inpatients with full privileges. Participants were screened on the phone and excluded if they had been hospitalized within the past 12 months for angina pectoris, myocardial infarction, congestive heart failure, or cardiac surgery of any kind; or currently had uncontrolled hypertension (i.e., blood pressure >140 systolic/90 diastolic). Additionally, participants were excluded if they met *DSM-IV-TR* criteria for current substance dependence or abuse within the past 3 months. Diagnosis and substance dependence/abuse were confirmed after consent was obtained using the Mini-International Neuropsychiatric Interview [MINI; (44)]. Using Donner and Eliaskiw’s (45) minimum sample size guidelines for examining reliability, a sample of 37 participants was determined to have 80% power to detect a significant difference between a minimum ICC standard of 0.60 and the expected level of 0.80, at α = 0.05. This sample size would allow for 15% oversampling based on previous PA and reliability testing research within the schizophrenia population (46).

### HAPA inventory piloting

To ensure comprehension, relevance, and suitability, the HAPA inventory underwent a brief qualitative screening and item refinement with 12 adult outpatient participants (8 males, 4 females, *M*_age_ = 52.2 ± 8.4 years) prior to reliability testing. All participants had a diagnosis of schizophrenia (*n* = 9) or schizoaffective disorder (*n* = 3), met the aforementioned inclusion/exclusion criteria for the reliability study, and gave informed consent to participate. Research ethics approval was obtained from the Centre for Addiction and Mental Health (CAMH) in Toronto, ON, Canada; Southlake Regional Health Centre (SRHC) in Newmarket, ON, Canada; and through the University of Toronto. Participants provided verbal feedback on drafts of the inventory during a 90-min interview with a trained research assistant (Markus Duncan) in a designated meeting room. Feedback was assessed using a content analysis framework and incorporated into the inventory. Participants received $30 compensation for participating in the inventory piloting stage.

### Measures

#### Participant screening and characteristics

After obtaining written consent to participate, competence to consent was verified by the research assistant verbally using the MacArthur Competence Assessment Tool for Clinical Research [MacCAT-CR; (47)]. The MINI was then administered to confirm diagnosis, followed by administration of the 18-item, anchored version of Brief Psychiatric Rating Scale [BPRS; (48)], and the severity scale of the Clinical Global Impression Scale [CGI-S; (49)] to assess symptom severity. Participants completed the 12-item Short Form Health Survey [SF-12; (50)], and the 18-item Apathy Evaluation Scale [AES; (51)], which assess self-reported quality of life and amotivation, respectively. The SF-12 is a common measure of physical and mental health that does not target a specific clinical population. The two norm-based (*M* = 50 ± 10) scales derived from the SF-12 – the Physical and Mental Health Composite Scores – each range from 0 to 100, with 100 being the highest level of health. The AES is scored from 18 to 72, with 72 representing the highest level of apathy (the lowest amount of goal-directed behavior). Participants also self-reported age, sex, height, weight, living arrangements (e.g., independent, with family), employment status, marital status, educational attainment, smoking habits, approximate date of schizophrenia onset, and current prescribed medications. Waist circumference was measured at the umbilicus.

Participants’ stage of PA engagement was determined using a single item question previously used in a sample with serious mental illness (12). Participants were asked to identify which of the following five statements is closest to how they feel about doing moderate-intensity PA: (1) I’m not physically active and I don’t intend to start (pre-contemplation); (2) I’m not physically active but I’m thinking about starting (contemplation); (3) I’m physically active once in-a-while but not regularly (preparation); (4) I’m physically active regularly but started only in the past 6 months (action); (5) I’m physically active regularly and have been so for longer than 6 months (maintenance).

#### Physical activity behavior

Participants’ self-reported moderate-to-vigorous intensity PA behavior over the past 7-days was assessed using the short form version of the International Physical Activity Questionnaire [IPAQ; (52)]. The IPAQ has previously been validated as a measure of PA behavior for adults with schizophrenia (46). In the current study, the IPAQ was modified to reflect the current Canadian Physical Activity Guidelines for Adults by including brisk walking as a form of moderate-intensity PA, while removing the item related to time spent walking.

#### HAPA inventory

The HAPA inventory consisted of 9 sections and 11 scales. Each item was rated on a seven-point scale with anchors varying according to the content of the scales. All scales targeted performing at least 150 min of PA of at least moderate-intensity over the next week as the outcome. The inventory is available in the Supplementary Material.

*Risk perceptions* were assessed with two subscales tapping into the vulnerability aspect of susceptibility – chance and likelihood. For the chance subscale, participants rated their chances of developing CVD, obesity, and type 2 diabetes in the future, each using a separate seven-point scale (1 = *very unlikely*; 4 = *moderately likely*; 7 = *very likely*). Meanwhile, the likelihood subscale asked participants to indicate the likelihood of them developing CVD, obesity, and type 2 diabetes in the future, using a seven-point scale (1 = *not at all strong*; 4 = *moderately strong*; 7 = *very strong*). Both subscales have been used in previous research to examine the relation between disease and psychological health risk and PA behavior among individuals with a physical disability (53). During the inventory pilot, participants had difficulty responding to the risk perception items if they already had a condition listed. Thus, participants in the reliability study whom already had the conditions identified were instructed to write “AH” for “already have” instead of using the rating scale.

*Affective outcome expectancies* were assessed based on Ajzen’s (54) recommendations for examining affective outcomes, and participant feedback during the HAPA inventory piloting stage. Given the concerns with amotivation among individuals with schizophrenia (55), the affective component of outcome expectancies was the target in the current study. Participants responded to the phrase, “For me, engaging in at least 150 min per week of PA of at least moderate-intensity over the next week would be…” using seven adjective pairs (boring–interesting, painful–not painful, unenjoyable–enjoyable, unpleasant–pleasant, exhausting–energizing, not fun–fun, distressing–calming). Participants indicated their agreement with the anchors of each pair using a seven-point scale (1 = *completely agree with the word on the left*; 7 = *completely agree with the word on the right*).

*Task self-efficacy* was measured using a single, six-item scale based on McAuley and Mihalko’s (56) guidelines for assessing task self-efficacy. Participants rated how confident they were on a seven-point scale (1 = *not confident at all*; 4 = *neutral*; 7 = *completely confident*) in their physical ability to do 10, 20, 30, 40, 50, and 60 min of at least moderate-intensity PA in one session without stopping if they were motivated enough to do so. Prior to the inventory piloting stage, moderate and vigorous PA were assessed as separate scales as suggested by Bandura (21). However, overwhelmingly, participants had difficulty remembering the distinction between moderate and vigorous PA and as a result, difficulty responding to two separate questions. To compensate, these separate scales were collapsed into a single scale assessing “at least moderate-intensity PA,” which remains congruent with the current Canadian Physical Activity Guidelines for Adults (43).

*Behavioral intentions* were measured using two items (54) that are commonly used in the PA domain [e.g., Ref. (57)]. Participants were asked to rate (1) how true the statement: “I will try to do at least 150 min per week of at least moderate-intensity PA over the next week” is for them (1 = *definitely false*; 7 = *definitely true*); and (2) to what extent is the statement: “I intend to do at least 150 min per week of at least moderate-intensity PA over the next week” likely for them (1 = *extremely unlikely*; 7 = *extremely likely*).

*Action planning* was assessed with five items (33) that have been used in previous HAPA research within the cardiac patient population. Participants rated (1 = *strongly disagree*; 4 = *neutral*; 7 = *strongly agree*) whether they had made detailed plans regarding their PA in terms of: (a) where; (b) when; (c) what types of activities they will do; (d) how often; and (e) how long they will engage in PA each time they are active.

*Coping planning* was assessed with five items using the same scale anchors as action planning. Participants rated whether they had made detailed plans about: (a) what to do if something interferes with their plans to do PA; (b) how to overcome setbacks to their PA plans; (c) how to stick with their intentions even in difficult situations; (d) how to overcome feeling poorly due to medication when they had made plans to engage in PA; and (e) how to keep engaging in PA once they start. These five items were based on Schwarzer’s (58) recommendations for measuring coping plans, in addition to the feedback obtained from the participants in the inventory piloting stage.

*Maintenance self-efficacy* measured participants’ confidence in their ability to participate in PA of at least moderate-intensity for at least 150 min over the next week even if they had to overcome a certain barrier. Fifteen barriers were identified based on previous research within the schizophrenia population (10) and feedback from participants during the HAPA inventory piloting stage. Examples of these barriers include not feeling well, lacking social support, and having difficulty making habits. All items were rated on a seven-point scale [1 = *not confident at all*; 4 = *neutral*; and 7 = *completely confident*; (21)].

*Recovery self-efficacy* measured participants’ confidence in their ability to do the following: (a) anticipate problems that might interfere with adding PA to their weekly schedule; (b) develop solutions to cope with potential obstacles that can interfere with adding PA; (c) resume PA the following week if a day of PA is interrupted; (d) resume PA if it is interrupted for a week or more; (e) identify key factors that trigger breaks in weekly PA; (f) learn to view occasional breaks in weekly PA as normal; and (g) learn to view occasional breaks in weekly PA as challenges to overcome rather than failures (59). All items were rated on a seven-point scale [1 = *not confident at all*; 4 = *neutral*; and 7 = *completely confident*; (21)].

*Action control* assessed participant’s awareness of their PA behavior and their use of self-regulatory strategies (e.g., self-monitoring). Participants indicated how true (1 = *definitely false*; 7 = *definitely true*) each of the following six statements were for them: (1) I constantly monitor whether I engage in PA of at least moderate-intensity often enough; (2) I am careful to ensure that I am active for at least 30 min at an intensity that is at least moderate each Time I engage in PA; (3) My PA program is often on my mind; (4) I am constantly aware of my PA program; (5) I really try to engage in PA of at least moderate-intensity regularly; (6) I try my best to meet my own standards for being physically active (60).

### Procedures

Research ethics approval was obtained from the Centre for Addiction and Mental Health (CAMH) in Toronto, ON, Canada; Southlake Regional Health Centre (SRHC) in Newmarket, ON, Canada; and through the University of Toronto. Participants were referred to the study through nurses, psychiatrists, and other studies at CAMH and SRHC. All sessions were completed at either CAMH or SRHC in a designated meeting room. Participants received $20 compensation per session ($40 total) for participating in the retest reliability study.

Participants attended two sessions, 1 week apart (Time 1 and 2, respectively) with a trained research assistant (Markus Duncan). Wherever possible, the sessions were scheduled to start at the same time of day. During the first session, all screening and characteristic information were collected, and participants responded to the stages of change question. During both sessions participants completed the HAPA inventory and the IPAQ. On average, the first session lasted between 45 and 90 min, while the second session lasted between 30 and 45 min.

#### Analysis

All statistical analyses were completed using IBM’s SPSS 22.0. Responses to items on the two risk perceptions subscales indicating that the participant already had the listed health condition were treated as missing data. Total minutes of moderate-to-vigorous intensity PA were summed separately at both time points from the IPAQ. Values ≥3 SDs from the mean were removed as outliers (61). Internal consistency was measured for all scales using Cronbach’s alpha with a 95% confidence interval. Test–retest reliability was measured for each scale using the bivariate Pearson correlation (*r*) between the mean scale scores at each time point. As a preliminary test of criterion validity, mean scores for all scales at Time 1 were correlated with minutes of at least moderate-intensity PA at Time 2, while correlations between scales at Time 1 were examined along relevant paths within the HAPA model.

## Results

### Inventory piloting stage

During the piloting stage, minimal changes were required to the original HAPA scales and primarily consisted of improving the visual layout of the inventory and rewording questions to reduce complexity in order to ensure consistent comprehension. In particular, participants had difficulty responding to the two risk perceptions subscales when they already had a condition, hence the “Already Have” option was added. As well, specific examples provided in the action planning section (e.g., “at a fitness center”) were removed as this caused participants to routinely ask how they should respond if their plans did not align with the example provided. Items were also added to the maintenance self-efficacy and coping planning scales based on participant feedback about barriers to PA they experience such as feeling slow/lethargic, or poorly.

### Participant characteristics

Table 1 provides a detailed description of the sample characteristics. A total of 28 outpatients were recruited and gave informed consent. Two participants were excluded after giving consent due to meeting criteria for substance dependence when assessed by the MINI. One participant canceled the retest session and was unable to reschedule due to unforeseen work obligations. As a result, data from 26 participants (16 males, 10 females, *M*_age_ = 41.5 ± 13.5 years) were used to assess internal reliability from the first session, while data from 25 participants were used for all outcomes related to the second session. Participants were generally representative of the larger outpatient schizophrenia population at CAMH, exhibiting symptom severity scores ranging from 2 (borderline) to 6 (severely ill) on the CGI-S and 21–59 on the BPRS, moderate-to-high apathy scores on the AES, and high rates (54%) of obesity. Two-tailed, one-sample *z*-tests revealed that both the mean Physical and Mental Health Composite Scores of the SF-12 were significantly different from the standardized general population mean (Physical Health Composite: *z* = −3.88, *p* < 0.001; Mental Health Composite: *z* = −7.50, *p* < 0.001). Table 1 describes the sample in detail.

**Table 1:** **Summary of participant demographics (*N* = 26)**.

Demographics	Value
Male:female	16:10
Age
Mean (SD)	41.5 (13.45)
Range	19–64 years old
Diagnosis
Schizophrenia	21
Schizoaffective	5
Symptom severity
BPRS mean score (SD)	33.4 (10.6)
CGI mean score (SD)	3.2 (1.2)
AES mean score (SD)	29.6 (8.7)
SF-12
Mean physical health composite score (SD)	42.4 (5.3)
Mean mental health composite score (SD)	35.3 (5.5)
BMI
Mean (SD)	32.4 (8.9)
Normal weight (BMI < 25)	4
Overweight	7
Obese (BMI > 30)	14
Waist circumference (*N* = 22)
Mean (SD)	112 cm (19 cm)
Physical activity levels
Mean (SD) at Time 1	288.0 min (332.6 min)
Mean (SD) at Time 2	192.6 min (169.9 min)
Stages of change (physical activity)
Pre-contemplation	2
Contemplation	6
Preparation	4
Action	4
Maintenance	10
Ethnicity
White	17
African	6
South Asian	1
Biracial	2
Employment
Not employed	15
Student	6
Part-time	4
Self-employed	1
Education
Some high school (no diploma)	3
Some high school (no diploma) with some postsecondary	3
High school diploma	6
Postsecondary education	14
Marital status
Single	23
Married	1
Separated	1
Divorced	1
Smoking habits
Current smokers	12
Mean (SD) cigarettes/day	14.6 (6.2)
Mode cigarettes/day	20

*Values are counts unless otherwise specified*.

### Scale internal consistency and test–retest reliability

Table 2 summarizes internal consistency, test–retest reliability, and correlation with minutes of moderate-to-vigorous PA for each scale of the HAPA inventory. The vast majority of HAPA scales demonstrated good internal consistency (αs > 0.80), and test–retest reliability (*r*s > 0.80), except for the two risk perceptions subscales. For the chance risk perceptions subscale, internal consistency was low at Time 1 (α = 0.62), respectively, while test–retest was good (*r* = 0.87). Meanwhile, the risk perceptions likelihood scale had acceptable internal consistency at both time points (αs = 0.75–0.77), but demonstrated low test–retest reliability (*r* = 0.21).

**Table 2:** **Summary of HAPA inventory psychometric properties and correlation with minutes of at least moderate physical activity**.

Scale	Time	*n*	Mean (SD)	Cronbach’s alpha (95% confidence interval)^a^	Test–retest reliability (*r*)^b^	Correlation (*r*) with PA^c^
Risk perceptions (chance)	1	25	3.36 (1.55)	0.62 (0.19–0.84) (*n* = 19)	0.87^f^ (*n* = 24)	−0.07 (*n* = 23)
	2	25	3.08 (1.63)	0.84 (0.65–0.93) (*n* = 19)	
Risk perceptions (likelihood)	1	26	3.92 (1.71)	0.75 (0.47–0.90) (*n* = 19)	0.21	0.12
	2	25	4.70 (1.69)	0.77 (0.53–0.90) (*n* = 21)	
Affective outcome expectancies	1	26	4.48 (1.33)	0.80 (0.67–0.90)	0.85^f^	0.35^d^
	2	25	4.53 (1.54)	0.91 (0.83–0.95)	
Task self-efficacy	1	26	5.31 (1.77)	0.96 (0.92–0.98)	0.96^f^	0.41^e^
	2	25	5.32 (1.80)	0.95 (0.90–0.97)	
Behavioral intentions	1	26	4.83 (2.04)	0.96 (0.90–0.98)	0.83^f^	0.38^d^
	2	25	4.80 (2.03)	0.98 (0.94–0.99)	
Action planning	1	26	4.72 (1.72)	0.92 (0.85–0.96)	0.88^f^	0.33
	2	25	4.72 (1.79)	0.93 (0.87–0.96)	
Coping planning	1	26	4.05 (1.63)	0.89 (0.80–0.94)	0.86^f^	0.32
	2	25	3.96 (1.60)	0.90 (0.82–0.95)	
Maintenance self-efficacy	1	26	4.28 (1.65)	0.97 (0.94–0.98)	0.85^f^ (*n* = 22)	0.40^e^
	2	25	4.07 (1.40)	0.96 (0.92–0.98)	
Recovery self-efficacy	1	26	4.66 (1.64)	0.94 (0.89–0.97)	0.91^f^	0.36^d^
	2	25	4.52 (1.58)	0.93 (0.88–0.97)	
Action control	1	25	4.67 (1.72)	0.89 (0.81–0.95)	0.91^f^ (*n* = 23)	0.41^e^ (*n* = 23)
	2	24	4.42 (1.68)	0.92 (0.86–0.96)	

*^a^*n* is the value listed indicated in the appropriate column unless otherwise indicated*.

*^b^*n* = 25 unless otherwise indicated*.

*^c^*n* = 24 unless otherwise indicated*.

*^d^Correlation is significant at the 0.10 level (two-tailed)*.

*^e^Correlation is significant at the 0.05 level (two-tailed)*.

*^f^Correlation is significant at the 0.01 level (two-tailed)*.

#### Correlations between the motivational HAPA stage constructs and intentions

Figure 1 illustrates cross-sectional bivariate correlations of the Time 1 pre-motivational HAPA factors with intentions. Notably, affective outcome expectancies (*r* = 0.72, *p* < 0.001) and task self-efficacy (*r* = 0.75, *p* < 0.001) exhibited significant, large-sized correlations with intentions to engage in moderate-to-vigorous intensity PA. Non-significant, small-sized correlations were exhibited between the two risk perceptions subscales and behavioral intentions (chance: *r* = −0.18, *p* = 0.40; likelihood: *r* = 0.15, *p* = 0.47).

#### Correlations between the volitional HAPA stage constructs and PA behavior

Bivariate correlations between the Time 1 volitional HAPA constructs and Time 2 moderate-to-vigorous intensity PA behavior are shown in Figure 1. Small to moderate-sized correlations were shown between moderate-to-vigorous intensity PA behavior and all of the HAPA volitional constructs (*r*s = 0.32–0.41), with significant correlations exhibited between PA behavior and action control and maintenance self-efficacy (*p*s = 0.05). The correlation between PA behavior and both behavioral intentions and recovery self-efficacy approached statistical significance (*p*s < 0.09).

## Discussion

The present study entails the first phase of a larger research program directed toward testing the utility of the HAPA framework for predicting objectively measured PA behavior among individuals with schizophrenia. Overall, the findings from the current study provide support for the internal consistency and test–retest reliability of a HAPA inventory that was pilot-tested for its relevancy and suitability for administration among individuals with schizophrenia. Examination of the internal consistencies of the HAPA scales revealed adequate to excellent indices of reliability for all scales, except the chance and likelihood risk perceptions subscales. Furthermore, significant, large-sized correlations were found for the 1-week test–retest reliability on all scales, except the likelihood risk perceptions subscale. One of the issues raised during both the inventory piloting stage and the reliability testing was that many of the participants already had the health conditions mentioned within the two risk perceptions subscales (i.e., CVD, obesity, and type 2 diabetes). Given that the schizophrenia population is at a high-risk for developing CVD (62, 63), it is not surprising that many of our participants already had these health complications. To address this issue, participants who had any of the three health complications listed were instructed to record that they “already had” the aforementioned condition, and to continue with the next item. We recommend that further research that uses these two risk perceptions subscales within the schizophrenia population incorporate the phrase “*developing or continuing to have* CVD/obesity/type 2 diabetes” within the scale item to enable participants who currently have one of the conditions mentioned to respond to the item, and perhaps, improve the internal consistency of these two subscales. Taken together, the reliability findings suggest that our modified HAPA inventory is a promising tool that, with continued development of the wording used within the risk perceptions subscales, can be used to further our understanding of the role of the HAPA variables in the prediction and promotion of PA within the schizophrenia population.

In addition to the encouraging findings for the HAPA scale reliabilities, preliminary support was demonstrated for the criterion validity of our modified HAPA inventory. For the motivational stage constructs, large, significant correlations were exhibited between Time 1 measures of task self-efficacy and affective outcome expectancies with intentions to engage in moderate-to-vigorous intensity PA behavior. Meanwhile, small to moderate-sized correlations were shown between the Time 1 measures of the volitional HAPA constructs and Time 2 moderate-to-vigorous intensity PA behavior. These findings are consistent with the HAPA tenets (17) and PA research among cardiac rehabilitation patients (23), which have found large-sized correlations between behavioral intentions and both task self-efficacy and outcome expectancies, and small to moderate-sized correlations between the volitional constructs and self-reported PA behavior. Further validation work is underway by our research team to determine whether these findings can be extended to a larger sample of individuals with schizophrenia.

Interestingly, the correlations between task self-efficacy and behavioral intentions (*r* = 0.75) and affective outcome expectancies (*r* = 0.72) and behavioral intentions were similar in magnitude. According to the HAPA tenets (16, 17), outcome expectancies and task self-efficacy both play influential roles in the prediction of behavioral intentions, while risk perceptions are considered to be more of a “distal antecedent” to the formation of behavioral intentions. However, Bandura’s social cognitive theory (21) postulates that self-efficacy has a stronger influence on behavioral intentions than outcome expectancies. Given the small sample size, further research is required to determine the most significant HAPA-based predictors of intentions to engage in moderate-to-vigorous intensity PA among individuals with schizophrenia. Identifying such predictors will assist with future development of effective, theory-based intentions for the promotion of moderate-to-vigorous intensity PA within this population.

Despite this being the first-ever, theory-based PA inventory developed for individuals with schizophrenia, there are some study limitations that must be acknowledged. First, the self-report PA measure that was used in this study may have resulted in some participants over-reporting their participation in moderate-to-vigorous intensity PA behavior. Similarly, the self-report nature of the other instruments used in this study may have resulted in some shared methods variance, and therefore significant correlations among the measured constructs, due to the influence of the common origin of the data. As previously mentioned, our research team is in the process of further validating the HAPA instrument using a more objective measure of PA behavior (i.e., accelerometers) among a larger sample of individuals with schizophrenia, which will reduce these aforementioned biases. This work will also allow for a more accurate depiction of PA levels within the schizophrenia population. Second, our sample size of 26 participants was lower than the projected sample of 37 we had intended to recruit to allow for 80% power to detect significant differences from a Cronbach’s α of 0.60. Consequently, some of the internal consistency analyses may have been underpowered, particularly those with a 95% confidence interval that include 0.60. The correlational analyses for the retest reliability and validity may be underpowered as well. Lastly, given the high Cronbach alphas for the majority of the scales, future research using the current HAPA instrument may consider reducing the number of items for each of the theorized constructs examined in order to maintain a more parsimonious inventory to assess PA within the schizophrenia population.

Overall, our findings provide preliminary support for the reliability and validity of the first-ever, HAPA inventory for examining predictors of moderate-to-vigorous intensity PA intentions and behavior among individuals with schizophrenia. Further validation research with this inventory using an objective measure of PA behavior will provide additional support for its psychometric properties within the schizophrenia population. This is an important and necessary first step in developing effective interventions to promote PA in this population.

## Conflict of Interest Statement

The authors declare that the research was conducted in the absence of any commercial or financial relationships that could be construed as a potential conflict of interest.

## Supplementary Material

The Supplementary Material for this article can be found online at http://www.frontiersin.org/Journal/10.3389/fpsyt.2014.00068/abstract
