# Supplementary material for: Development and Reliability Testing of a Health Action Process Approach Inventory for Physical Activity Participation among Individuals with Schizophrenia
Source: Front Psychiatry. 2014 Jun 10;5:68. doi: 10.3389/fpsyt.2014.00068 (PMC4051131; doi:10.3389/fpsyt.2014.00068)
Supplement: Supplementary file 1 [file Data_Sheet1.PDF]

Section 1: Motivational Phase

Risk Perceptions

1. Using the scale below, please choose the most appropriate response for each question.

a) My chances of developing or continuing to have *cardiovascular disease* in the future are...

1	2	3	4	5	6	7
Very unlikely			Moderately likely			Very likely

b) My chances of developing or continuing to have *obesity* in the future are…

1	2	3	4	5	6	7
Very unlikely			Moderately likely			Very likely

c) My chances of developing or continuing to have *type 2 diabetes* in the future are...

1	2	3	4	5	6	7
Very unlikely			Moderately likely			Very likely

The next set of questions ask about engaging in physical activities of **at least moderate intensity**.

Moderate intensity physical activity refers to activities where you can talk, but not sing your favourite song, during the activity. With these activities you are working hard enough to raise your heart rate. On a scale relative to an individual's personal capacity, moderate intensity physical activity is usually a 5 or 6 on a scale of 0 to 10 (where 0 is resting and 10 is extremely hard).

Examples include walking briskly (~ 3 miles/hr or 5 km/hr), water aerobics, bicycling casually, and tennis. More intense activities include race walking, jogging, running, swimming laps, and heavy gardening (continuous digging or hoeing).

This type of activity **does not** include walking casually to get somewhere.

Remember that you may refer back to these descriptions if you need examples later in the questionnaire.

Affective Outcome Expectancies

For each **pair** of words use the scale to indicate which word best represents how you would complete the sentence below. A “**1**” represents that you completely agree with the word on the **left** and “**7**” represents that you completely agree with the word on the **right**.

For me, engaging in at least 150 minutes per week of physical activity of at least moderate intensity over the next week would be…

**Boring**	1	2	3	4	5	6	7	**Interesting**
**Painful**	1	2	3	4	5	6	7	**Not Painful**
**Unenjoyable**	1	2	3	4	5	6	7	**Enjoyable**
**Unpleasant**	1	2	3	4	5	6	7	**Pleasant**
**Exhausting**	1	2	3	4	5	6	7	**Energizing**
**Not Fun**	1	2	3	4	5	6	7	**Fun**
**Distressing**	1	2	3	4	5	6	7	**Calming**

Task Self-Efficacy

For the following questions, please indicate how confident you are on a scale from 1 (not confident at all) to 7 (completely confident).

Assuming you were very motivated, how confident are you that you could physically do the following amounts of physical activity of **at least moderate intensity** in one session without stopping **over the next week.**

Please answer for each amount of time.

	**Not Confident At All**			**Neutral**			**Completely Confident**
**10 minutes**	1	2	3	4	5	6	7
**20 minutes**	1	2	3	4	5	6	7
**30 minutes**	1	2	3	4	5	6	7
**40 minutes**	1	2	3	4	5	6	7
**50 minutes**	1	2	3	4	5	6	7
**60 minutes**	1	2	3	4	5	6	7

Behavioural Intentions

a) To what extent is the following statement true for you? "**<u>I will try</u>** to do at least 150 minutes per week of at least moderate intensity physical activity over the next week."

1	2	3	4	5	6	7
Definitely False						Definitely True

b) To what extent is the following statement likely: "**<u>I intend</u>** to do at least 150 minutes per week of at least moderate intensity physical activity over the next week."

1	2	3	4	5	6	7
Extremely Unlikely						Extremely Likely

Section 2: Volitional Phase

The next set of questions ask about engaging in physical activities of **at least moderate intensity**.

Moderate intensity physical activity refers to activities where you can talk, but not sing your favourite song, during the activity. With these activities you are working hard enough to raise your heart rate. On a scale relative to an individual's personal capacity, moderate intensity physical activity is usually a 5 or 6 on a scale of 0 to 10 (where 0 is resting and 10 is extremely hard).

Examples include walking briskly (~ 3 miles/hr or 5 km/hr), water aerobics, bicycling casually, and tennis. More intense activities include race walking, jogging, running, swimming laps, and heavy gardening (continuous digging or hoeing).

This type of activity **does not** include walking casually to get somewhere.

Remember that you may refer back to these descriptions if you need examples later in the questionnaire.

Action Planning

INSTRUCTIONS: The following items relate to your physical activity that is **at least moderate intensity** **over the next week**. Using the scales provided, please rate the extent to which you agree with each statement.

1. I have made detailed plans about **WHERE** I will do physical activity that is at least moderate intensity over the next week.

1	2	3	4	5	6	7
Strongly disagree			Neutral			Strongly Agree

2. I have made detailed plans about **WHEN** I will do physical activity that is at least moderate intensity over the next week.

1	2	3	4	5	6	7
Strongly disagree			Neutral			Strongly Agree

3. I have made detailed plans about **WHAT TYPES OF ACTIVITIES** I will do over the next week.

1	2	3	4	5	6	7
Strongly disagree			Neutral			Strongly Agree

INSTRUCTIONS: The following items relate to your physical activity that is **at least moderate intensity** over the next week. Using the scales provided, please rate the extent to which you agree with each statement.

4. I have made detailed plans about **<u>HOW OFTEN</u>** I will do physical activity that is at least moderate intensity over the next week.

1	2	3	4	5	6	7
Strongly disagree			Neutral			Strongly Agree

5. I have made detailed plans about **<u>HOW LONG</u>** I will do physical activity that is at least moderate intensity over the next week **each time I am active**.

1	2	3	4	5	6	7
Strongly disagree			Neutral			Strongly Agree

Coping Planning

INSTRUCTIONS: The following items relate to your physical activity that is **at least moderate intensity** **over the next week.** Using the scales provided, please rate the extent to which you agree with each statement.

1. I have made detailed plans about **WHAT TO DO IF SOMETHING INTERFERES** with my plans to do physical activity of at least moderate intensity over the next week.

1	2	3	4	5	6	7
Strongly disagree			Neutral			Strongly Agree

2. I have made detailed plans about **HOW TO COPE WITH SETBACKS** in my plans to do physical activity of at least moderate intensity over the next week.

1	2	3	4	5	6	7
Strongly disagree			Neutral			Strongly Agree

3. I have made detailed plans about **HOW TO STICK TO MY INTENTIONS** to do physical activity of at least moderate intensity over the next week, even in difficult situations.

1	2	3	4	5	6	7
Strongly disagree			Neutral			Strongly Agree

INSTRUCTIONS: The following items relate to your physical activity that is **at least moderate intensity** over the next week. Using the scales provided, please rate the extent to which you agree with each statement.

4. I have made detailed plans about **HOW TO OVERCOME FEELING POORLY** due to my medication when I had planned to engage in physical activity of at least moderate intensity.

1	2	3	4	5	6	7
Strongly disagree			Neutral			Strongly Agree

5. I have made detailed plans about how to **KEEP ENGAGING IN PHYSICAL ACTIVITY** of at least moderate intensity once I start.

1	2	3	4	5	6	7
Strongly disagree			Neutral			Strongly Agree

Maintenance Self-Efficacy
Assuming you were very motivated, how confident are you that you will participate in physical activity that is **at least moderate intensity for at least 150 minutes per week <u>over the next week</u>**…

a) …even if it takes you a long time to make it a habit?

1	2	3	4	5	6	7
Not Confident At All			Neutral			Completely Confident

b) …even if you are worried and troubled?

1	2	3	4	5	6	7
Not Confident At All			Neutral			Completely Confident

c) …even if you don't see success at once?

1	2	3	4	5	6	7
Not Confident At All			Neutral			Completely Confident

d) …even if you are tired?

1	2	3	4	5	6	7
Not Confident At All			Neutral			Completely Confident

Assuming you were very motivated, how confident are you that you will participate in physical activity that is **at least moderate intensity for at least 150 minutes per week <u>over the next week</u>**…

e) …even if you are stressed out?

1	2	3	4	5	6	7
Not Confident At All			Neutral			Completely Confident

f) …even if you feel tense?

1	2	3	4	5	6	7
Not Confident At All			Neutral			Completely Confident

g) …even if you lack social support?

1	2	3	4	5	6	7
Not Confident At All			Neutral			Completely Confident

h) …even if you feel slow?

1	2	3	4	5	6	7
Not Confident At All			Neutral			Completely Confident

Assuming you were very motivated, how confident are you that you will participate in physical activity that is **at least moderate intensity for at least 150 minutes per week over the next week**…

i) …even if you have to start all over again several times until you succeed?

1	2	3	4	5	6	7
Not Confident At All			Neutral			Completely Confident

j) …even if your partner or family isn't physically active?

1	2	3	4	5	6	7
Not Confident At All			Neutral			Completely Confident

k) …even if you don't feel well?

1	2	3	4	5	6	7
Not Confident At All			Neutral			Completely Confident

l) …even if you don't know where to go to engage in physical activity?

1	2	3	4	5	6	7
Not Confident At All			Neutral			Completely Confident

Assuming you were very motivated, how confident are you that you will participate in physical activity that is **at least moderate intensity for at least 150 minutes per week over the next week…**

m) …even if you don't know what types of physical activity to do?

1	2	3	4	5	6	7
Not Confident At All			Neutral			Completely Confident

n) …even if you are physically sore or in pain?

1	2	3	4	5	6	7
Not Confident At All			Neutral			Completely Confident

o) …even if it would cost you money?

1	2	3	4	5	6	7
Not Confident At All			Neutral			Completely Confident

Recovery Self-Efficacy

<u>Over the next week</u>, how confident are you that you can:

a) Anticipate problems that might interfere with you adding **physical activity of at least moderate intensity** to your weekly schedule?

1	2	3	4	5	6	7
Not Confident At All			Neutral			Completely Confident

b) Develop solutions to cope with potential obstacles that can interfere with you adding **physical activity of at least moderate intensity** to your weekly schedule?

1	2	3	4	5	6	7
Not Confident At All			Neutral			Completely Confident

c) Resume your physical activity the following week if a <u>day</u> of **at least moderate intensity physical activity** is interrupted?

1	2	3	4	5	6	7
Not Confident At All			Neutral			Completely Confident

<u>Over the next week</u>, how confident are you that you can:

d) Resume your **physical activity of at least moderate intensity** when it is interrupted for a week or more?

1	2	3	4	5	6	7
Not Confident At All			Neutral			Completely Confident

e) Identify key factors that trigger breaks in your weekly physical activity **of at least moderate intensity**?

1	2	3	4	5	6	7
Not Confident At All			Neutral			Completely Confident

f) Learn to view occasional breaks to your weekly **physical activity of at least moderate intensity** as normal?

1	2	3	4	5	6	7
Not Confident At All			Neutral			Completely Confident

<u>Over the next week</u>, how confident are you that you can:

g) Learn to view breaks in your **physical activity of at least moderate intensity** as challenges to overcome rather than failures?

1	2	3	4	5	6	7
Not Confident At All			Neutral			Completely Confident

Action Control

To what extent are the following statements true for you?

1. I constantly monitor whether I engage in physical activity of at least moderate intensity often enough.

1	2	3	4	5	6	7
Definitely False						Definitely True

2. I am careful to ensure that I am active for at least 30 minutes at an intensity that is at least moderate each time I engage in physical activity.

1	2	3	4	5	6	7
Definitely False						Definitely True

3. My physical activity program is often on my mind**.**

1	2	3	4	5	6	7
Definitely False						Definitely True

To what extent are the following statements true for you?

4. I am constantly aware of my physical activity program.

1	2	3	4	5	6	7
Definitely False						Definitely True

5. I really try to engage in physical activity of at least moderate intensity regularly.

1	2	3	4	5	6	7
Definitely False						Definitely True

6. I try my best to meet my own standards for being physical active.

1	2	3	4	5	6	7
Definitely False						Definitely True
